# Shallow water seeding cultivation enhances cold tolerance in tobacco seedlings

**DOI:** 10.1186/s12870-024-05422-9

**Published:** 2024-07-24

**Authors:** Xuan Tao, Lei Yang, Mingfa Zhang, Yangyang Li, Hanqian Xiao, Lingyi Yu, Chaowei Jiang, Zeyu Long, Yiyang Zhang

**Affiliations:** 1 College of Agronomy, Hunan Agricultural University, Changsha, China; 2 Hunan Research Institute of Tobacco Science, Changsha, China; 3 Hunan Provincial Tobacco Corporation, Changsha, China; 4 Xiangxi Branch of Hunan Provincial Tobacco Corporation, Xiangxi, China

**Keywords:** Tobacco, Cold stress, Float system, Cold tolerance

## Abstract

Cold stress can impact plant biology at both the molecular and morphological levels. We cultivated two different types of tobacco seedlings using distinct seeding methods, observing significant differences in their cold tolerance at 4 °C. After 12 h cold stress, shallow water seeding cultivation treatment demonstrates a relatively good growth state with slight wilting of the leaves. Tobacco grown using the float system exhibited short, thick roots, while those cultivated through shallow water seeding had elongated roots with more tips and forks. After cold stress, the shallow water seeding cultivation treatment demonstrated higher antioxidant enzyme activity, and lower malondialdehyde (MDA) content.

Transcriptome analysis was performed on the leaves of these tobacco seedlings at three stages of cold treatment (before cold stress, after cold stress, and after 3 days of recovery). Upon analyzing the raw data, we found that the shallow water seeding cultivation treatment was associated with significant functional enrichment of nicotinamide adenine dinucleotide (NAD) biosynthesis and NAD metabolism before cold stress, enrichment of functions related to the maintenance of cellular structure after cold stress, and substantial functional enrichment related to photosynthesis during the recovery period. Weighted gene co-expression network analysis (WGCNA) was conducted, identifying several hub genes that may contribute to the differences in cold tolerance between the two tobacco seedlings. Hub genes related to energy conversion were predominantly identified in shallow water seeding cultivation treatment during our analysis, surpassing findings in other areas. These include the AS gene, which controls the synthesis of NAD precursors, the PED1 gene, closely associated with fatty acid β-oxidation, and the RROP1 gene, related to ATP production.

Overall, our study provides a valuable theoretical basis for exploring improved methods of cultivating tobacco seedlings. Through transcriptome sequencing technology, we have elucidated the differences in gene expression in different tobacco seedlings at three time points, identifying key genes affecting cold tolerance in tobacco and providing possibilities for future gene editing.

**Supplementary Information:**

The online version contains supplementary material available at 10.1186/s12870-024-05422-9.

## Introduction

Cold stress poses a significant challenge in abiotic stress conditions [1]. Most crops in tropical and subtropical regions are highly sensitive to cold stress [2]. Cold stress reduces the activity of the enzymes involved in photosynthesis [3], leading to a decrease in the production of energy-rich compounds such as ATP and NADPH [4]. Exposure to cold stress affects the fluidity of cell membranes, resulting in the leakage of ions and other substances from the cells [5, 6]. This disruption of membrane integrity can lead to cell death, impacting plant growth and yield [7]. Over evolutionary time, plants have developed various mechanisms to adapt to low temperatures, including the C-repeat binding factor (CBF) regulatory pathway, also known as the dehydration responsive element binding pathway [8].

In South China, sudden cold weather, often referred to as "spring frost," damages float system tobacco seedlings, negatively impacting tobacco quantity and quality [9]. Minimizing the impact of cold stress on crop yield and quality has been a major focus in agricultural science. Studies have shown that the application of exogenous substances can enhance plant cold tolerance. For example, tomato seedling roots treated with 1 mM H_2_O_2_ for 1 h exhibited enhanced cold tolerance [10]. Melatonin has also been found to enhance antioxidant properties in plants at low temperatures [11], while Ca^2+^ plays a crucial role in plant cold response [12]. However, implementing these measures in actual agricultural production is challenging due to cost constraints.

In China, tobacco seedling cultivation is primarily based on the floating seedling factory nursery, which produces uniform seedlings and facilitates seedling management. However, the float system's nutrient-rich environment can harbor harmful microorganisms, such as Pythium seedling blight, leading to rootstock rot infected by *P. myriotylum* [13]*.* Pesticide treatment is required to suppress this disease, which can have environmental implications [14]. Additionally, the root system in the float system exhibits oxytropism, developing towards high oxygen content rather than gravity, potentially resulting in a weak root system [15].

To optimize the cultivation process while retaining the advantages of the float system, a shallow water cultivation method has been explored in certain tobacco cultivation areas in China. Shallow water seeding cultivation controls the water level in the seedling pool. Because the nutrient solution in the pool is absorbed or evaporated, adding the nutrient solution at regular intervals ensures that the growth environment for tobacco seedlings alternates between wet, slightly dry, and wet cycles. This differs from the widely used float system, where tobacco seedlings remain in a constantly wet environment. The alternating wet-dry–wet cycle in shallow water seeding cultivation promotes better root development in tobacco seedlings. We believe that the shallow water seeding cultivation method saves water compared to the float system, and reduces the production of large amounts of harmful algae and microorganisms from the eutrophic nutrient solution in the nursery pool. However, the intermittent addition of nutrient solution increases the labor cost, which can be solved by mechanical automation in the future. Also, research has shown that tobacco seedlings cultivated using this method exhibit stronger cold resistance [16]. However, further research is needed to comprehensively compare the morphological and physiological differences between tobacco seedlings cultivated using the shallow water cultivation method and the float system, especially under cold stress. Subsequent genetic analysis and interpretation could provide valuable insights for optimizing tobacco seedling cultivation practices in China.

## Result

### Root system morphology

**Fig. 1:**
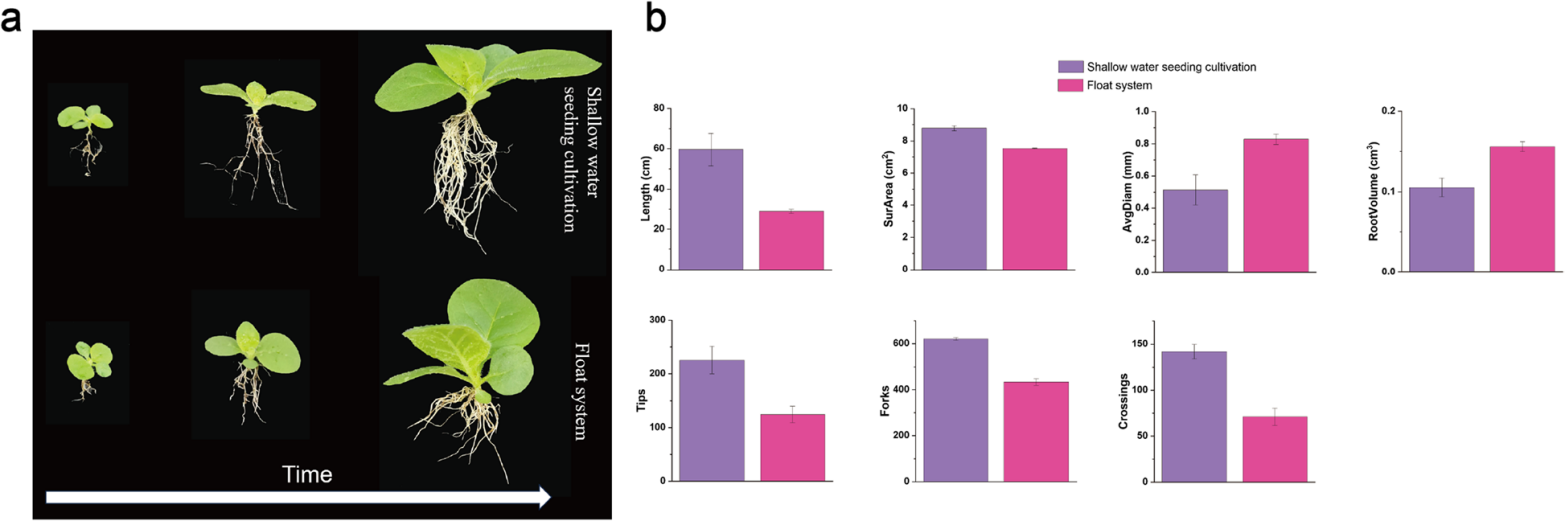
**a** From left to right are photos of the root system growth and development of the different treatments over time, the top group is shallow water seeding cultivation treatment, and the bottom group is float system treatment. **b** Root system phenotype of tobacco seedlings under different treatments at the 4-leaf 1-heart stage

The developmental timeline of tobacco seedlings cultivated through different seedling cultivation treatments is depicted in Fig. 1. In the top three photos, the shallow water seeding cultivation treatment shows noticeably slender and more numerous roots compared to the float system.

**Fig. 2:**
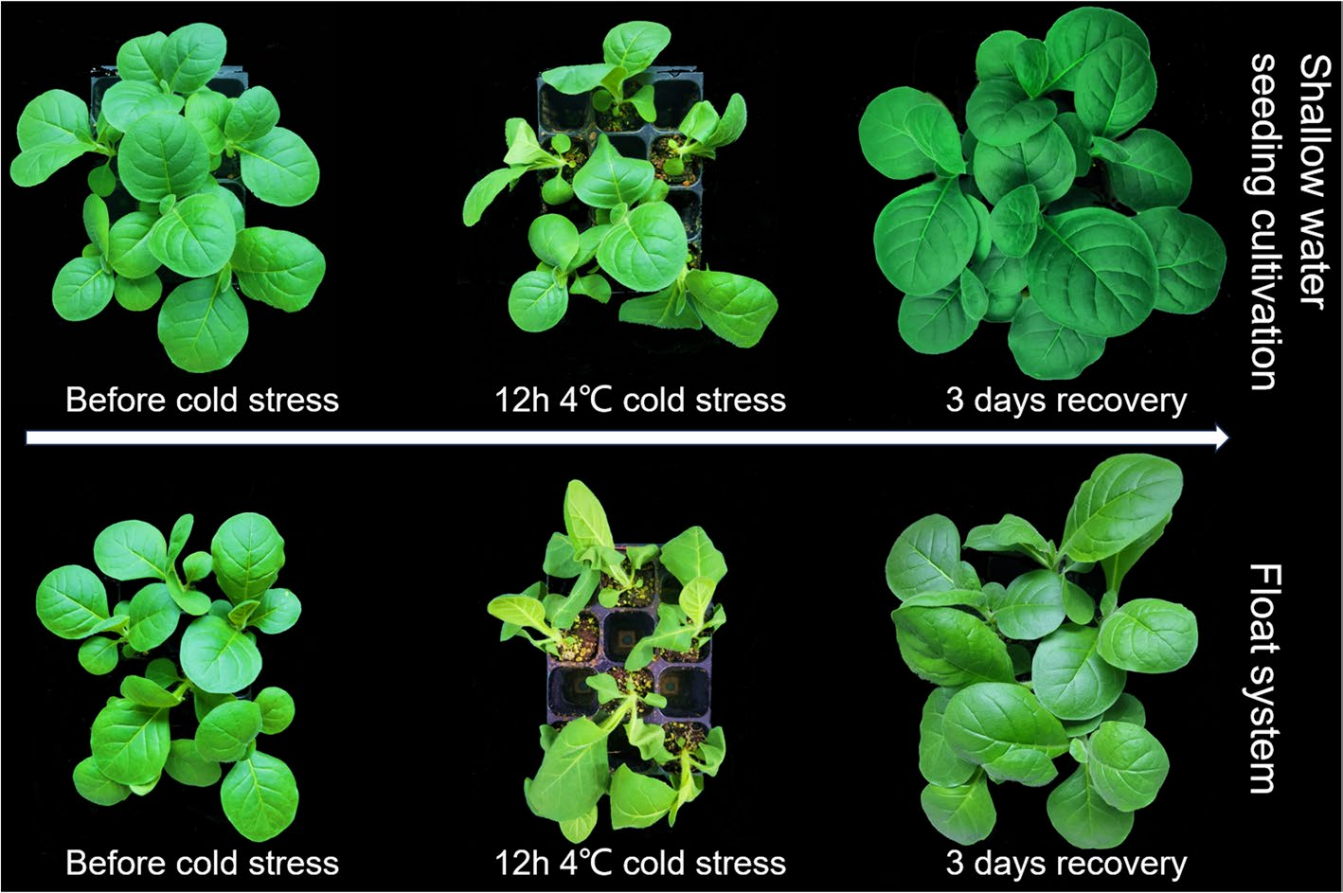
Phenotypic differences before and after cold stress in tobacco seedlings under different treatments. The upper three photographs are shallow water seeding cultivation treatment, the under three photographs is float system treatment

To further investigate the root system differences among the treatments, we utilized the WinRHIZO root analysis system to scan and analyze the root systems of tobacco seedlings at the 4-leaf 1-heart stage, leading to the bar graph shown in Fig. 2b. The shallow water seeding cultivation treatment exhibited a 106% increase in root length, a 16.84% increase in root surface area, an 81.5% increase in root tip number, and a 43.42% increase in root branching compared to the float system treatment. However, the shallow water seeding cultivation treatment showed a 38.06% decrease in root diameter and a 32.5% decrease in root volume compared to the float system treatment (Table S1).

These results suggest that the shallow water seeding cultivation treatment promotes the development of a more extensive and structurally complete root system, potentially enhancing nutrient absorption capabilities during growth and development.

### Tobacco seedings under 4℃ cold stress

After 12 h of cold stress at 4 °C, the shallow water seeding cultivation treatment exhibited less wilting compared to the float system treatment (Fig. 2). In the shallow water seeding cultivation treatment, only a few smaller leaves appeared wilted and crumpled, while almost all leaves in the float system treatment were wilted and crumpled, with some stalks even exhibiting bending.

Following three days of recovery in the greenhouse, both treatments were able to resume normal growth. However, the crumpling of the leaf edges in the float system treatment had not fully recovered, suggesting that the damage caused by cold stress to the float system may persist in the short term.

### Determination of physiological activity before and after cold stress

**Fig. 3:**
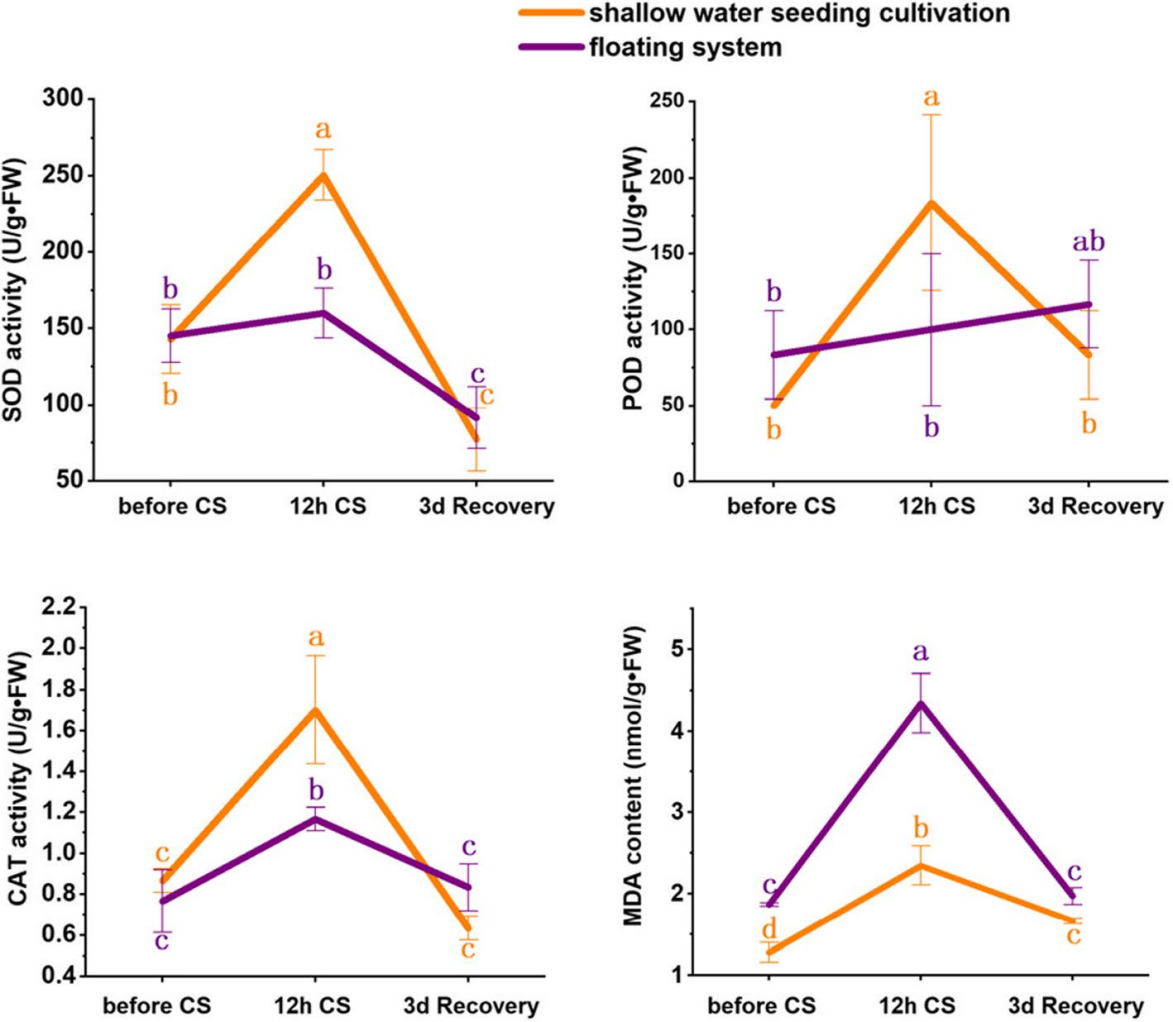
Physiological activity of tobacco seeding under different treatments before and after CS (cold stress). Based on ANOVA, the letters on the error bars imply significance between the data; a completely different letter means that the two data are significantly different at the 0.05 level, and containing the same letter means that the two data are not significantly different

Examination of physiological activity indices in tobacco seedlings from the two different treatments before and after cold stress revealed significant differences in all parameters (Fig. 3). Cold stress induces the production of reactive oxygen species (ROS) in plants, which can damage plant cells. Three key antioxidant enzymes in plants—superoxide dismutase (SOD), peroxidase (POD), and catalase (CAT)—showed elevated activities in both treatments after exposure to cold stress. Notably, the activities of these enzymes were higher in the shallow water seeding cultivation treatment after 12 h of cold stress at 4 °C, with increases of 56.78%, 83.33%, and 45.71% for SOD, POD, and CAT, respectively, compared to the float system treatment. Following 3 days of recovery in the greenhouse, antioxidant enzyme activities decreased in both treatments. Malondialdehyde (MDA), an indicator of membrane lipid peroxidation and cell membrane damage, sharply increased in the float system treatment after 12 h of cold stress, reaching a level 85.09% higher than that in the shallow water seeding cultivation treatment.

### Identification of differentially expressed genes (DEGs) among different treatments

To investigate the factors contributing to the variability in cold responsiveness among tobacco seedlings from different treatments, we conducted a parametric transcriptome analysis of tobacco leaves from two different seeding cultivation methods at three time points: before cold stress, after 12 h of cold stress, and after 3 days of recovery. We designated the shallow water seeding cultivation treatment as A and the float system treatment as B. This resulted in six treatments: A0 (shallow water seeding cultivation before cold stress), A1 (shallow water seeding cultivation after 12 h of cold stress), A2 (shallow water seeding cultivation recovery for 3 days), B0 (float system before cold stress), B1 (float system after 12 h of cold stress), and B2 (float system recovery for 3 days).

**Fig. 4:**
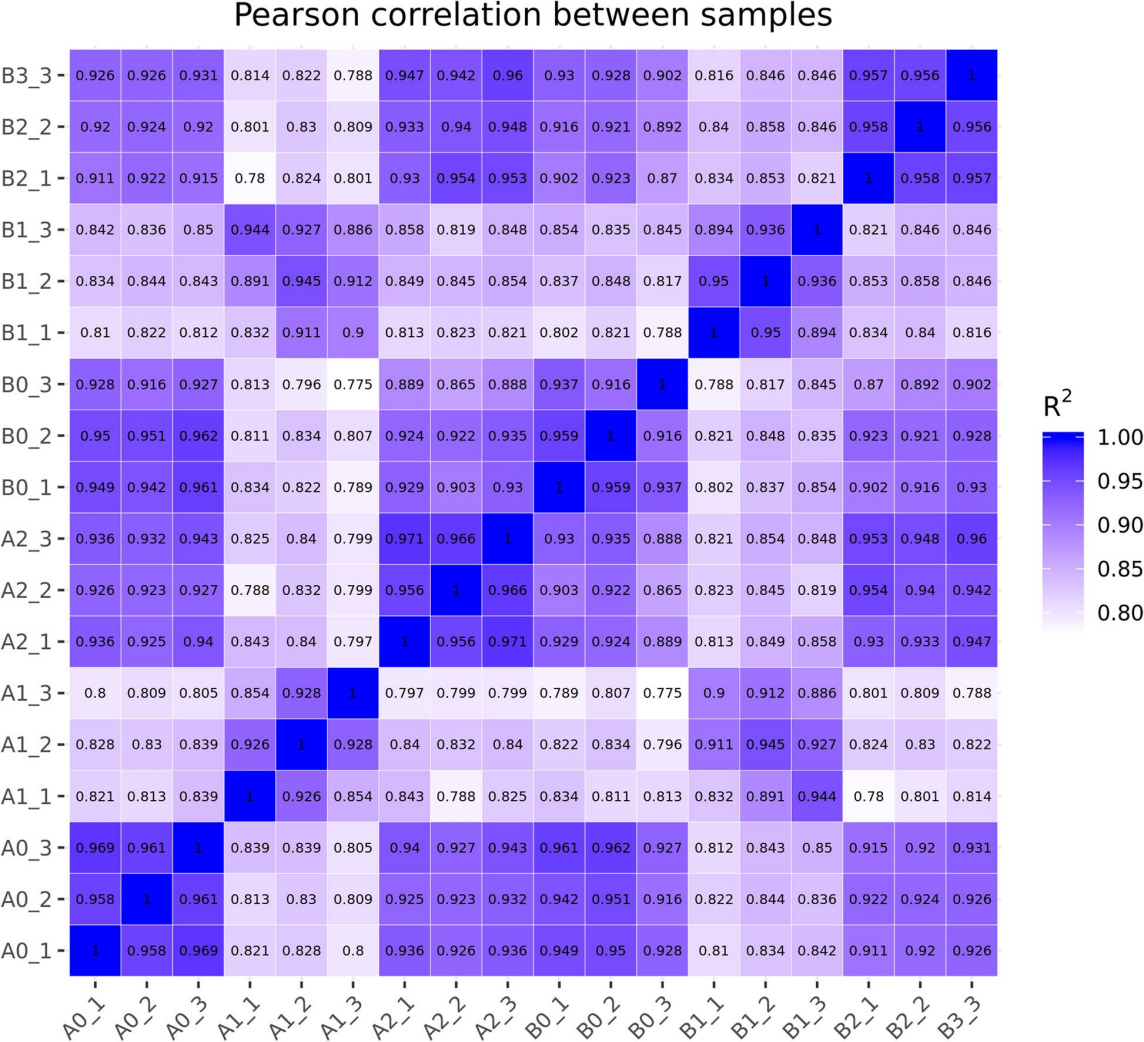
Pearson correlation between samples

Following data quality control and reference genome comparison, we conducted a Pearson correlation test on the samples to assess the correlation between replicates of the same sample (Fig. 4). We observed a strong correlation between replicates of the same sample. Significant changes were observed in samples A1 and B1 after cold stress compared to samples A0 and B0 before cold stress.

**Fig. 5:**
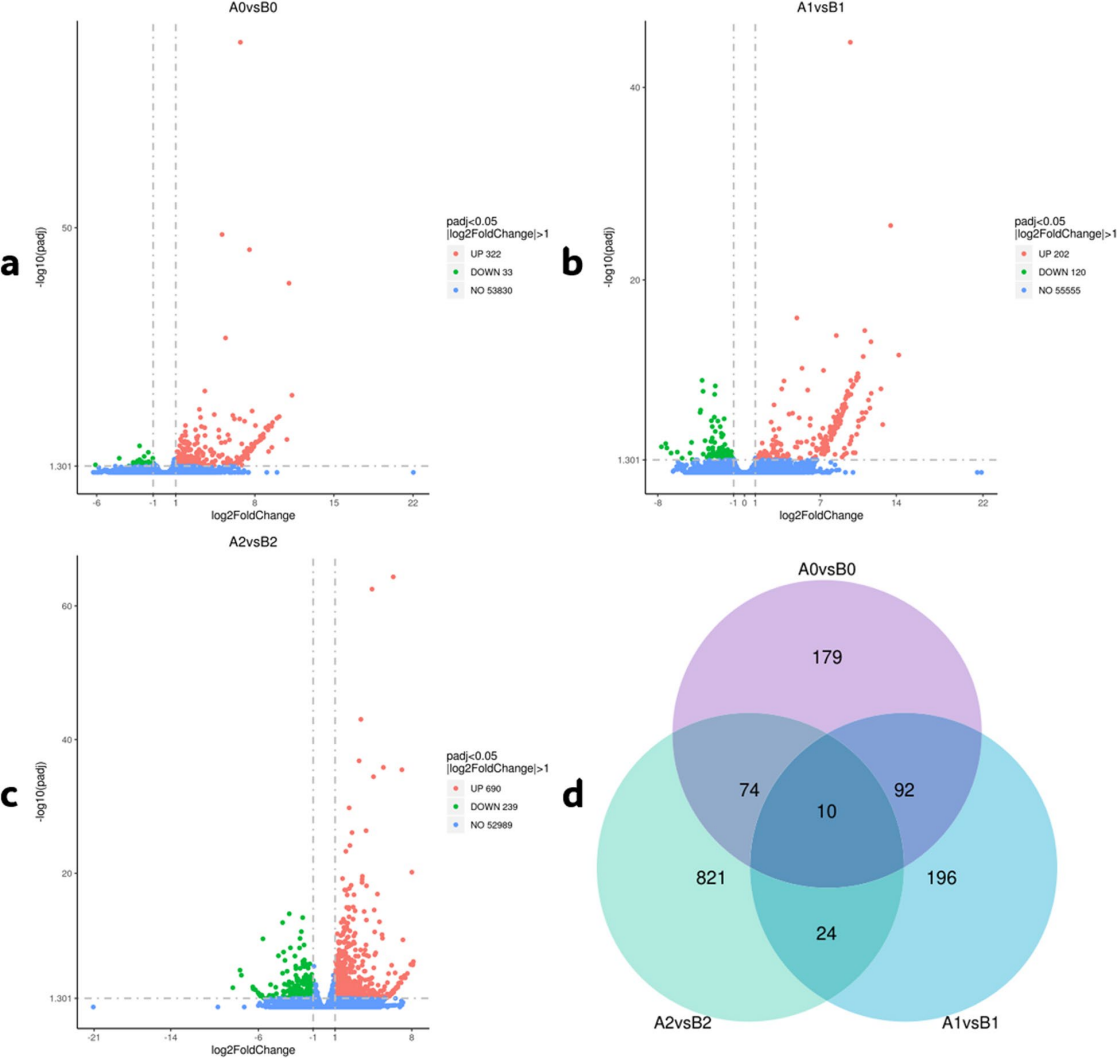
**a**, **b**, **c** Volcano of DEGs for different comparison combinations. **d** The Venn diagrams for the three comparative combinations

We conducted a quantitative analysis of differentially expressed genes (DEGs) between treatment combinations and identified a total of 355 DEGs between A0 and B0, with 322 up-regulated and 33 down-regulated genes (Fig. 5a). Between A1 and B1, there were 322 DEGs, with 202 up-regulated and 120 down-regulated genes (Fig. 5b). Additionally, between A2 and B2, there were 929 DEGs, with 690 up-regulated and 239 down-regulated genes (Fig. 5c). We observed more up-regulated DEGs in treatment A during the three phases of cold stress, with a substantial increase in DEGs between A2 and B2 (after 3 days of recovery). Venn diagrams for the three comparative combinations revealed that 196 DEGs were specific to A1vsB1, 179 DEGs were unique to A0vsB0, and 821 DEGs belonged to A2vsB2 (Fig. 5d). Furthermore, 10 DEGs were differentially expressed throughout the experiment. Some genes belonging to the AP2/ERF-ERF and bHLH families, as well as other genes related to low temperature in plants, were activated in the comparative combination A1vsB1 (Table S2), consistent with previous studies.

### GO and KEGG enrichment analysis

We plotted the DEGs of each treatment on a heatmap to visualize different expression pattern regions between treatments A and B at each low-temperature stage. The differential expression of genes in these regions may contribute to the difference in cold resistance between A and B.

**Fig. 6:**
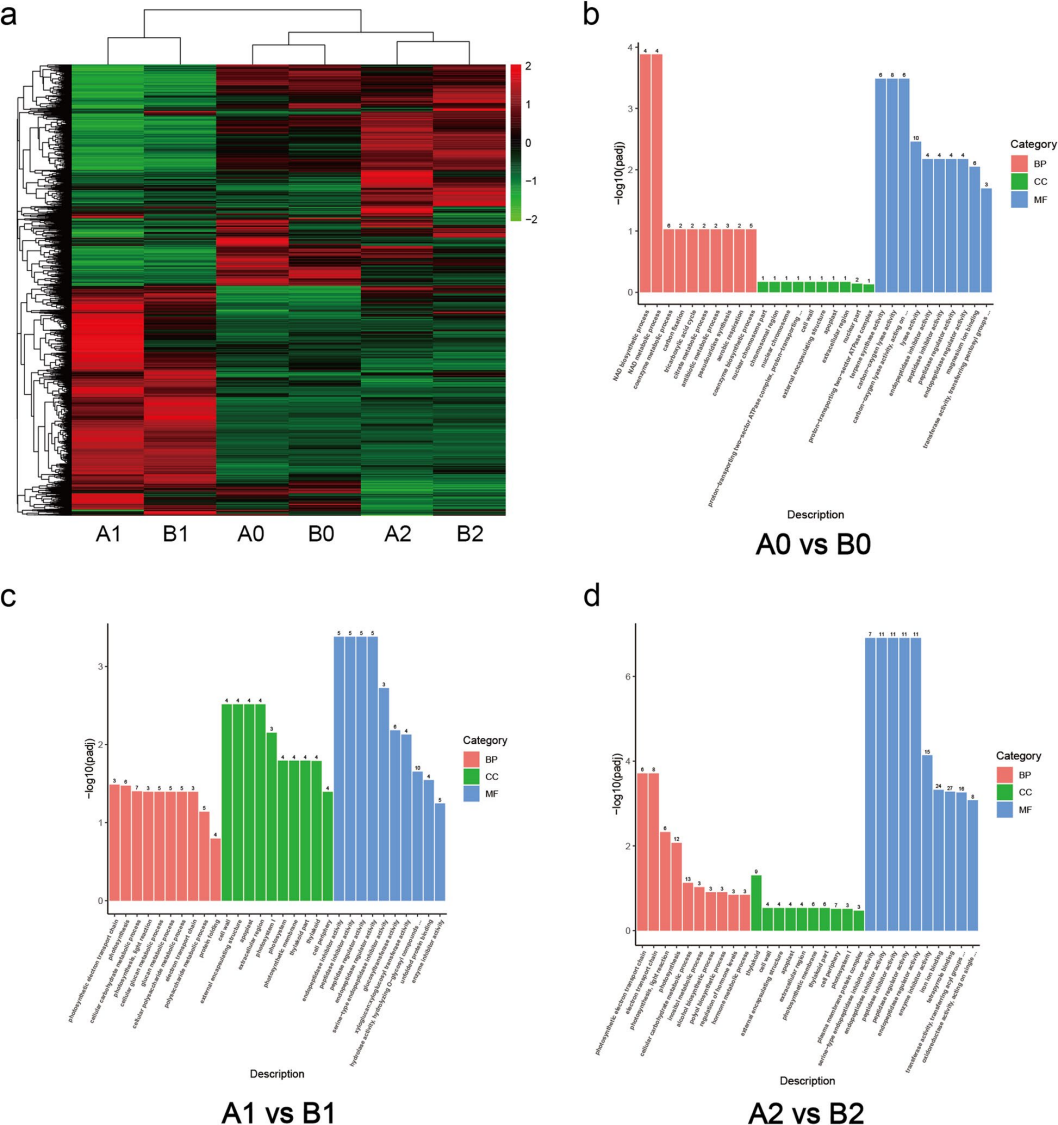
**a** Heatmap of DEGs for different treatments. The red color is the high expression, and the green color is the low expression. **b**, **c**, **d** GO analysis of different comparative combinations. The most significant 10 Terms for each function are selected, and a total of 30 Terms are plotted in a bar chart for display, or all Terms if there are fewer than 30

To elucidate the functional aspects of these DEGs affecting tobacco seedlings, we conducted GO and KEGG analyses. GO (Gene Ontology) is a database describing gene functions, including biological process (BP), cellular component (CC), and molecular function (MF). KEGG (Kyoto Encyclopedia of Genes and Genomes) integrates genomic, chemical, and systemic functional information. We generated histograms for the 30 most significant terms from the GO enrichment analysis results and scatter plots for the 20 most significant terms from the KEGG enrichment analysis results (Fig. 6).

The GO analysis of DEGs in the A0vsB0 comparative combinations revealed significant enrichment in BP terms such as NAD biosynthetic process, NAD metabolic process, and coenzyme metabolic process; in CC terms such as nuclear chromosome part; and in MF terms such as terpene synthase and carbon–oxygen lyase activity.

The GO analysis of DEGs in the A1vsB1 comparative combinations showed significant enrichment in BP terms such as photosynthetic electron transport chain, photosynthesis, and cellular carbohydrate metabolic process; in CC terms such as cell wall, external encapsulating structure, apoplast, and extracellular region; and in MF terms such as endopeptidase inhibitor activity, peptidase inhibitor activity, and peptidase regulator activity.

The GO analysis of DEGs in the A2vsB2 comparative combinations indicated significant enrichment in BP terms such as photosynthetic electron transport chain, electron transport chain, photosynthesis, and light reaction; in CC terms such as thylakoid and cell wall; and in MF terms such as serine-type endopeptidase inhibitor activity, endopeptidase inhibitor activity, peptidase inhibitor activity, and peptidase regulator activity.

**Fig. 7:**
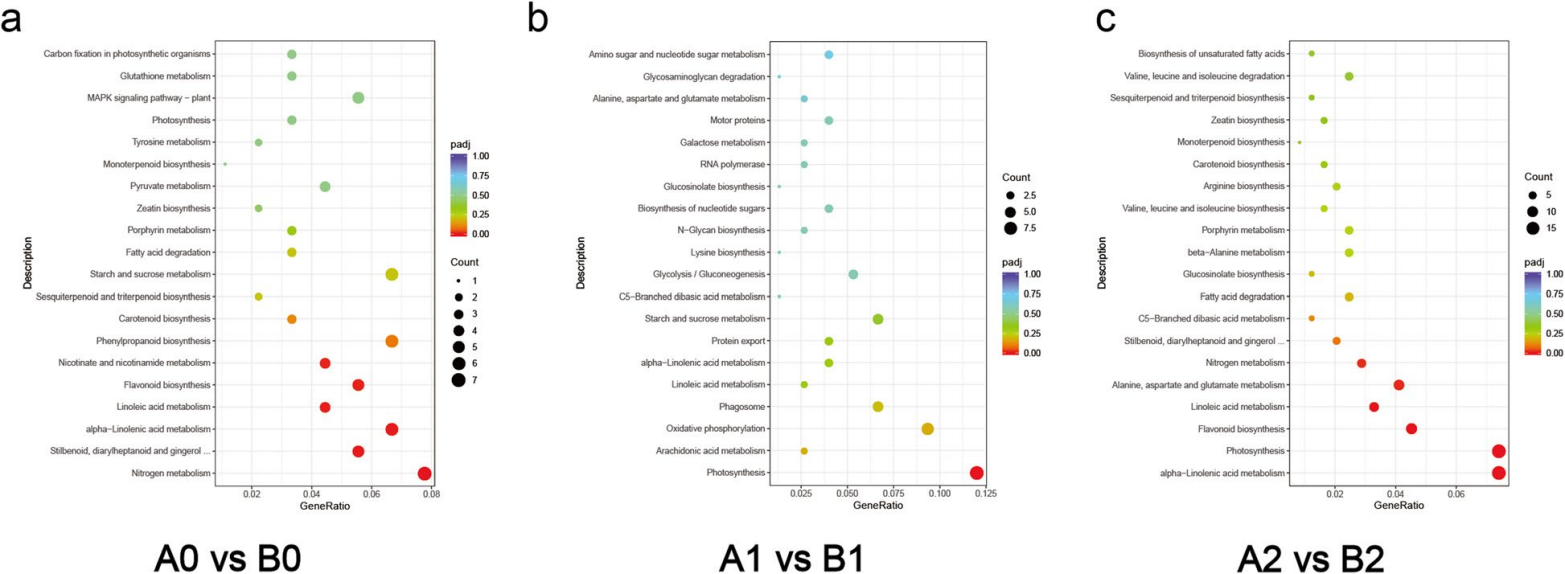
**a**, **b**, **c** KEGG analysis of different comparative combinations. Gene Ratio means the ratio of the number of differential genes annotated to KEGG pathway numbers to the total number of DEGs

In the A0vsB0 comparative combination, KEGG analysis revealed more DEGs enriched for functions such as nitrogen metabolism and flavonoid biosynthesis (Fig. 7a). In the A1vsB1 comparative combination, DEGs were significantly enriched in functions related to photosynthesis, oxidative phosphorylation, and phagosome (Fig. 7b). Interestingly, in the A2vsB2 comparative combination, DEGs were significantly enriched for functions related to alpha-Linolenic acid metabolism and photosynthesis (Fig. 7c).

Although cold stress occurs under dark conditions, studying the functional aspects of photosynthesis in A1vsB1 provides insights into the mechanisms involved in acclimating to cold stress. Furthermore, after 3 days of recovery (A2 vs. B2), all 18 genes related to photosynthesis showed positive enrichment in A2, indicating their importance in the recovery process following cold stress.

### Weighted gene co-expression network analysis

To further investigate the underlying reasons for the difference in cold tolerance between treatments A and B, we conducted weighted gene co-expression network analysis (WGCNA). This method allows for the grouping of genes with similar expression patterns into modules and the identification of hub genes.

**Fig. 8:**
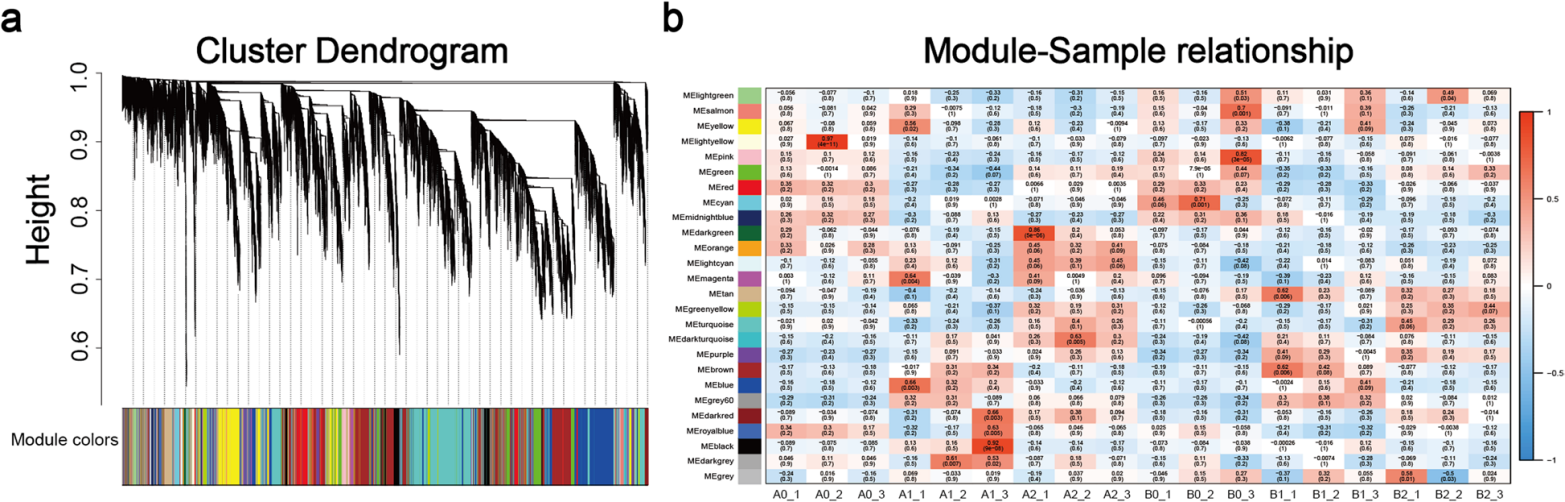
**a** The top part of the diagram is the gene clustering tree in the network, a leaf is a gene, and different gene modules are the branches of this tree. In the middle part, Dynamic Tree Cut is used to obtain different module graphs, in which different colors represent different modules. The bottom part of Merged colors is the graph of merged modules with dissimilarity coefficients less than 0.25, where different colors represent the merged modules. **b** Heatmap of module-sample correlation. The horizontal coordinate is the sample, the vertical coordinate is the module, and the number in each grid represents the correlation between the module and the sample; the closer this value is to 1, the stronger the positive correlation between the module and the sample; the closer it is to -1, the stronger the negative correlation between the module and the sample. The number in parentheses represents the significance *P* value, the smaller this value is, the stronger the significance is

Initially, we set a Pearson coefficient threshold between genes to define their co-expression relationships. To ensure the robustness of our analysis, correlation coefficients were raised to the power of N to avoid weak correlations near the threshold (Fig. S1). Subsequently, we constructed a clustering tree using the WGCNA R package, resulting in the identification of 26 modules represented by different colors (Fig. 8a).

The module-sample relationship (Fig. 8b) revealed intriguing patterns. The orange module exhibited higher expression levels in A0 and A2, with a negative correlation observed with B across all periods. Conversely, the tan module showed positive correlations with B0, B1, and B2 but not with A. Notably, during the 12-h cold stress period, the black module showed a strong positive correlation with A1, suggesting that genes within this module may play a crucial role in enhancing cold resistance in tobacco seedlings.

**Fig. 9:**
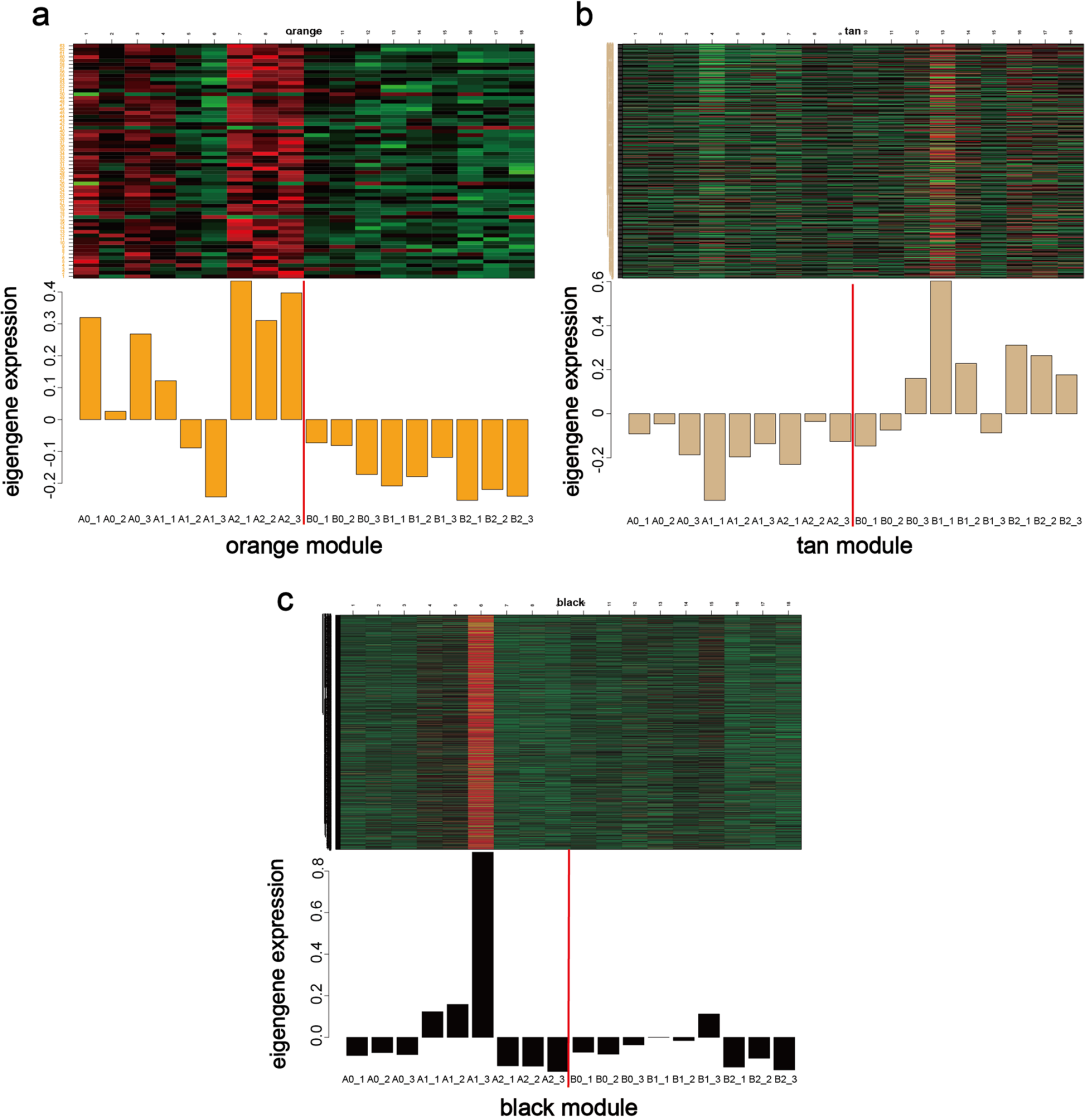
**a**, **b**, **c** Expression pattern of genes in the module, the result of this graph can be divided into two parts to view, the header comment is the name of the module, the left side of the upper graph is the name of the gene, and the horizontal coordinate is the name of the sample, the upper graph is the heatmap of the expression of the genes in the module in different samples, the red color is the high expression, and the green color is the low expression. The under figure shows the expression pattern of the module eigenvalues in different samples, and the horizontal coordinates are the sample names

To better visualize the relationship between the expression levels of these three modules, we constructed a graph depicting their gene expression patterns over time (Fig. 9). The eigengene expression of the orange module was notably higher in A before cold stress, decreased post-cold stress, and exhibited a rapid increase after 3 days of recovery. Intriguingly, this module was not expressed in B.

In contrast, the eigengene expression of the tan module was predominantly high in B, particularly after cold stress and 3 days of recovery. Conversely, the black module was unique among the 26 modules, showing expression only during the 12-h cold stress period. Additionally, the expression of the black module was significantly higher in A compared to B. These distinct gene expression patterns in the black module may underlie the observed differences in cold tolerance between A and B.

The number of genes within each module varies, with the orange module containing the fewest at 63, the tan module containing 552, and the black module containing the most with 1723 genes. Each gene's functional importance within a module, referred to as the hub gene, was determined based on its connectivity with other genes. A table of gene connectivity in each module was compiled (Table S3), with the K value indicating gene connectivity—larger values indicating greater connectivity. The k-Within value represents the connectivity of genes within the module, and the top-ranked genes based on k-Within values are considered hub genes.

**Fig. 10:**
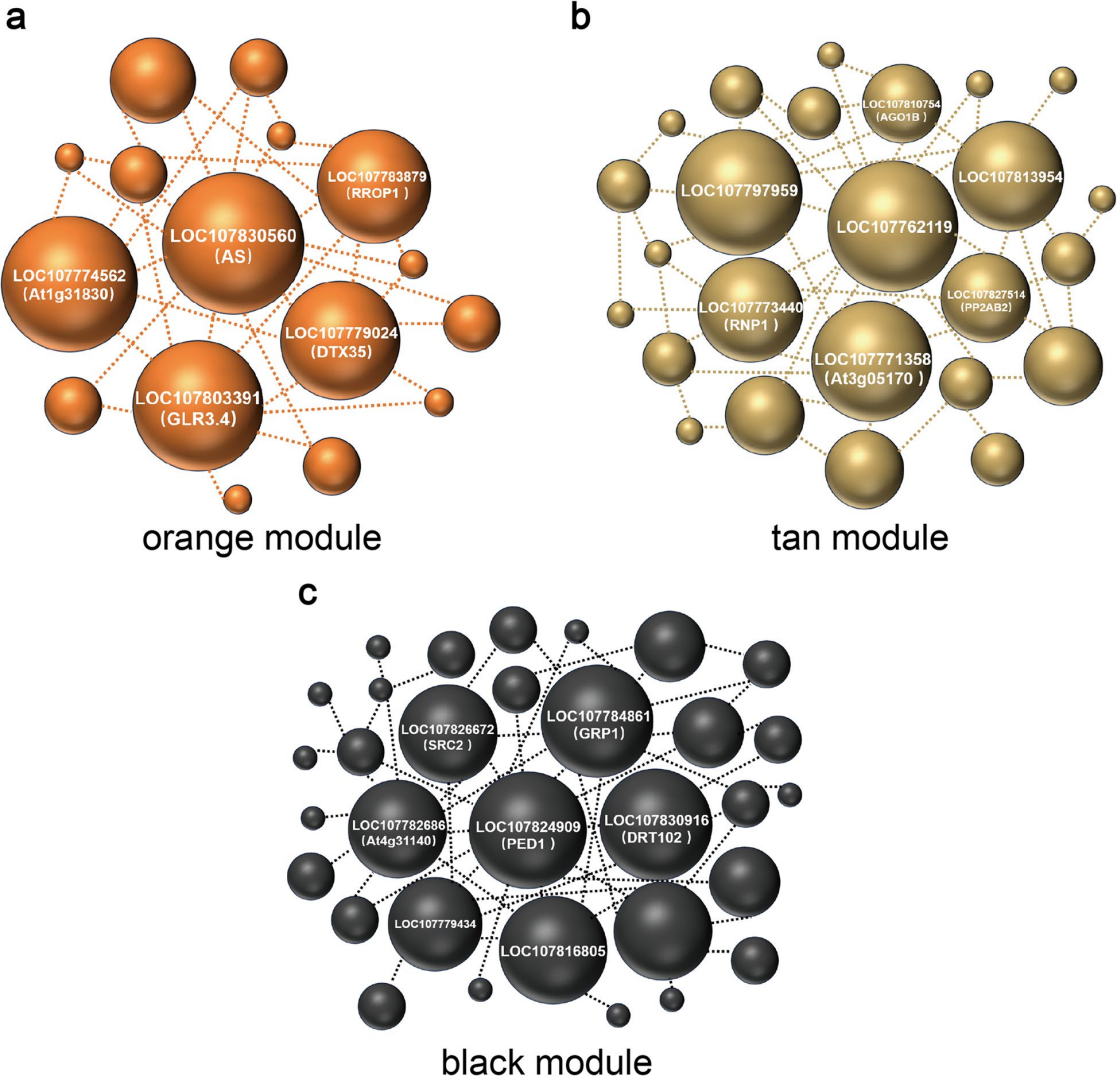
**a**, **b**, **c** Visualization of Gene Connectivity in Three Modules. The size of each gene corresponds to its k-Within value, with larger spheres indicating higher values. Gene names are labeled on the spheres based on their top k-Within values

The orange module, which is highly expressed only in A (shallow water seedling cultivation treatment), likely contains the hub gene responsible for the growth difference between treatments A and B. We identified 5 hub genes in the top 5 k-Within rankings in the orange module, including AS, At1g31830, GLR3.4, and DTX35 (Fig. 10a). These genes are known to function in adversity response, signaling, and toxicity release in other plants.

The tan module, which is highly expressed only in B (float system), also contains hub genes. Five of the top 10 hub genes in this module are uncharacterized, such as LOC107762119 and LOC107797959 (Fig. 10b). The other five genes, including At3g05170, are involved in metabolic pathways like glycolysis and RNA synthesis processes.

The black module, which shows expression only after cold stress, may contain key genes that respond to cold stress. The expression of genes in this module was higher in A than in B after cold stress, potentially contributing to the superior phenotype and antioxidant enzyme activities in A. Hub genes in the black module, ranked by k-Within values, include PED1, GRP1, DRT102, LOC107816805, and SRC2 (Fig. 10c). These genes are involved in processes such as fatty acid breakdown, genetic information maintenance, cellular structure stability, and cell membrane signaling.

**Fig. 11:**
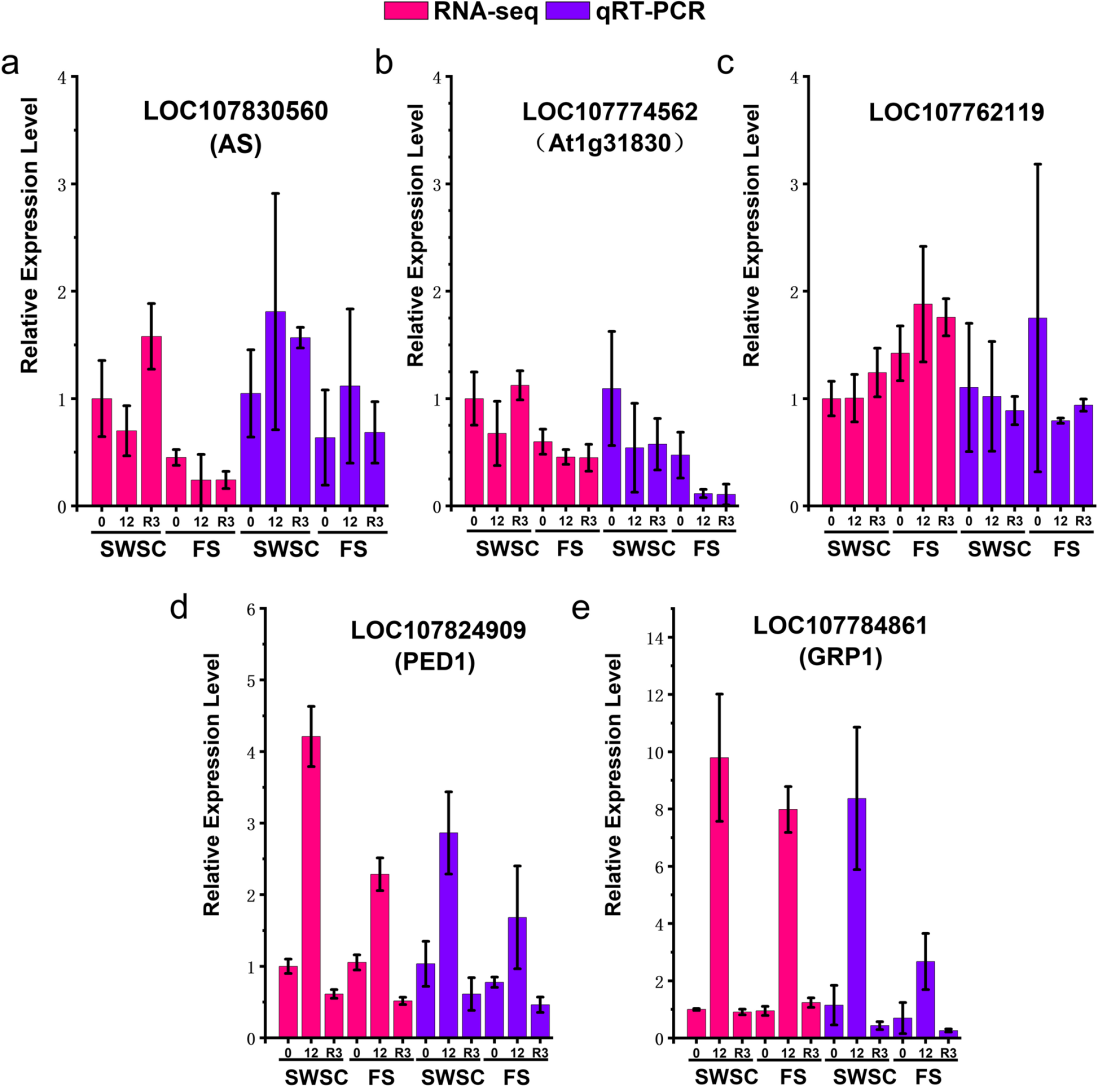
The results of qRT-PCR assay of selected genes were plotted in comparison with RNA-seq. The horizontal coordinate in the graph indicates the cold treatment time, SWSC stands for shallow water seeding cultivation, and FS stands for float system. we used the expression of the selected genes in the SWSC cold treatment for 0 h as 1 to normalize the relative expression of the selected genes in RNA-seq for other cold treatment times. The results of qRT-PCR assays were normalized to the expression level of the internal reference gene, Ntubc2, and transformed to represent the relative expression level of the selected gene in qRT-PCR using 2^−ΔΔCT^ for output operations. Three biological replicates were used for all assays

In order to verify the accuracy of the genes identified by WGCNA, we randomly selected 6 genes from each module with high gene connectivity for qRT-PCR, including 2 genes in the orange module (LOC107830560, LOC107774562), 1 gene in the tan module (LOC107762119), and 2 genes in the black module (LOC107824909, LOC107784861). We compared the qRT-PCR results with the expression of the corresponding genes derived from RNA-seq, and found that four of the five selected genes had similar trends in expression changes (Fig. 11). LOC107824909 (PED1), for example, exhibited trends at each time point that were identical to the results in RNA-seq (Fig. 11d). Only 12 h cold stress samples in LOC107830560 (AS) had high qRT-PCR results compared to RNA-seq (Fig. 11a). However, the discrepancy between the qRT-PCR results of LOC107762119 and the trend of changes exhibited in RNA-seq is large (Fig. 11c), which may be due to the changes in the samples during prolonged storage, and the possibility of gene false positives in RNA-seq analysis cannot be ruled out.

## Discussion

### Differences in root structure between the two treatments

In plants, apart from absorbing oxygen through their leaves, they also require oxygen and obtain nutrients from the soil through their roots [17]. In Method B, a considerable portion of the substrate's pores are filled with nutrient solution, leading to reduced oxygen availability within the substrate. This oxygen deficit can hinder the roots' energy production efficiency, potentially resulting in decreased nutrient uptake. When exposed to low oxygen levels, plant roots initially exhibit a cessation of lateral root elongation and formation [18]. In contrast, Method A involves a lower water depth, ensuring that the pores in the upper layer of the substrate remain unfilled with nutrient solution, thus maintaining sufficient oxygen levels. As depicted in Fig. 2b, Method A demonstrates superior performance in root length and root tip number, indicating that this cultivation method provides a more conducive oxygen environment for root growth.

A robust root system in plants can enhance resistance to cold stress in the aboveground parts [19]. Cold stress initially damages cell membranes, leading to membrane hardening. Subsequently, it reduces the activity of various protein enzymes in cells, impairing photosynthesis. Finally, it affects gene expression, disrupting normal protein synthesis [20]. Following 12 h of cold stress at 4 °C (Fig. 2), leaves in the shallow water cultivation method exhibited superior performance, indicating that different cultivation methods indeed affect cold tolerance.

### Differences in antioxidant capacity between the two treatments

To verify the difference of cold tolerance between two treatments, we measured the levels of three antioxidant enzymes and MDA in leaves subjected to cold stress at three time points. Cold stress induces the generation of reactive oxygen species (ROS) in plants. ROS production activates gene expression to protect plant cells from cold stress damage, but excessive ROS damages plant cells [21]. Plants have evolved mature antioxidant mechanisms over long periods of evolution. SOD, POD, and CAT are the main antioxidant enzymes in plants. SOD catalyzes the dismutation of superoxide radicals, producing H_2_O_2_ and O_2_. POD is involved in scavenging superoxide radicals and H_2_O_2_ in cells. CAT decomposes H_2_O_2_ into H_2_O and O_2_. MDA, as a product of membrane lipid peroxidation, can be used to evaluate the degree of membrane lipid damage in cells based on its content [22–25]. Method A showed higher antioxidant enzyme activity after cold stress (Fig. 4), indicating that this cultivation method may possess stronger antioxidant capabilities. The MDA content measurement also indicated lower levels in Method A, suggesting that this cultivation method may exhibit greater tolerance to cold stress.

### Expression of DEGs in the two treatments across three stages

To gain deeper insights into the molecular basis of the differential cold tolerance observed between these two treatments, we performed transcriptomic analysis on the leaves of tobacco (*Nicotiana tabacum*, cultivar *Xiangyan 7*) subjected to cold stress at three distinct time points. Given the limited availability of gene sequences for *Xiangyan 7* tobacco, we utilized the gene sequences of TN90 (Nicotiana tabacum), a closely related variety, as a reference. To more easily identify the patterns causing differences in cold tolerance, we combined the enrichment analysis of DEGs between treatments with WGCNA and analyzed the results.

Through transcriptomic analysis, we identified DEGs between the comparative treatments. Prior to cold stress (A0 vs B0), DEGs were enriched in GO functions related to NAD biosynthetic and metabolic processes. NAD is pivotal in the electron transfer chain of plant respiration, closely linked to nitrogen utilization [26]. Correspondingly, KEGG analysis revealed enrichment in nitrogen metabolism, suggesting potential differences in nitrogen utilization in respiration between A0 and B0. Furthermore, terpene synthase function was enriched in A0 vs B0, hinting at possible terpenoid production in A0 as a response to cold stress [27, 28], although further research is required for confirmation.

After 12 h of cold stress at 4 °C, the initial impact is on cell membrane lipids. Treatment B exhibited severe wilting and curling, while treatment A showed relatively minor damage. DEGs between A1 and B1 were enriched in GO functions related to cell wall, external encapsulating structures, apoplast, and the extracellular region, indicating a role in maintaining cell rigidity under cold stress. Additionally, we observed enrichment in numerous functions associated with peptidases, including both positive and negative enrichments. Peptidases and peptidase inhibitors play crucial roles in plant growth, development, and defense against external stresses, including abiotic stress such as cold stress [29].

Three days after recovery, both treatments A and B resumed normal growth and development. However, the leaf margin wrinkling observed in treatment B persisted (Fig. 2), indicating that the damage inflicted on the cells of B leaves by cold stress did not dissipate in the short term. In the GO analysis of the A2 vs B2 comparison group, significant enrichment was observed in functions related to the photosynthetic electron transport chain, electron transport chain, photosynthesis, light reaction, and thylakoid. Cold stress can uncouple photophosphorylation from electron transport, deactivate light-activated ATPases, and disrupt the osmotic balance of the thylakoid membrane [30, 31]. The positive enrichment of photosynthesis-related functions in treatment A2 suggests that its photosynthetic capacity is superior to that of treatment B2 following recovery.

WGCNA grouped DEGs with similar expression patterns into modules. A total of 26 modules were identified, among which the orange module showed high expression specifically in treatment A, the tan module showed high expression specifically in treatment B, and the black module showed high expression specifically after cold stress.

The Orange module, characterized by high expression exclusively in treatment A, likely contains hub genes responsible for the observed differences in cold tolerance between treatments A and B. Among these, the AS gene encodes an asparagine synthetase with a glutamine amido transferase domain [32]. It's noteworthy that in our previous GO analysis (Fig. 6), we observed significant enrichment of functions related to NAD biosynthetic process in A before cold stress. Asparagine serves as a critical precursor for NAD synthesis, the active state of the AS gene before cold stress in A may suggest active NAD synthesis. NAD, a crucial coenzyme in plants, participates in numerous redox reactions and plays an indispensable role in energy production [33]. This observation may imply a greater involvement of NAD in the energy conversion process in treatment A, potentially contributing to the differential performance of treatments A and B under cold stress conditions. Additionally, several hub genes related to stress responses were identified in the orange module, such as At1g31830, DTX35, and RROP1. Among these, At1g31830 is associated with ABA in *Arabidopsis thaliana* [34], DTX35 belongs to the detoxification efflux carrier family [35], and RROP1 is involved in ATP production in *Solanum tuberosum* [36].

The Black module exhibited differential expression between A and B, with higher expression levels observed in A compared to B (Fig. 9). Importantly, the genes within this module were expressed following exposure to cold stress, irrespective of whether it occurred in treatment A or B. This suggests that the Black module may contain hub genes that are activated in response to cold stress. Through analysis, the genes with the strongest connectivity in the Black module include PED1, GRP1, DRT102, LOC107816805, and SRC2. Among these, PED1 in *Arabidopsis thaliana* has been implicated in fatty acid β-oxidation and the glyoxylate cycle, encoding a 3-ketoacyl-CoA thiolase [37]. In the current experiment, tobacco was subjected to cold stress in dark conditions, causing the stalling of the photosynthetic system. Higher plants can convert fatty acids into glucose through gluconeogenesis to provide energy for normal cellular functions. The elevated expression of PED1 in A may suggest its fatty acid β-oxidation-related functions are stronger than those of B. GRP1 has been reported in *Craterostigma plantagineum* to be associated with apo plastic functions involved in cellular resistance to drought dehydration [38]. This correlates with the enrichment of apoplast functions observed in A1 vs B1 GO analysis (Fig. 6), this suggests that GRP1 may also influence apoplastic functions in treatment A during cold stress resistance. Cold stress can adversely affect the physical and chemical integrity of DNA [39], leading to genetic information disruption and affecting plant growth and development. Research has shown that Escherichia coli transformed with DRT102 from *Arabidopsis thaliana* exhibited significantly increased survival rates under UV radiation and displayed inheritable UV radiation resistance in offspring [40]. The abundant expression of DRT102 in A may also facilitate the repair of tobacco DNA damaged during cold stress. LOC107816805 and GRP1 both encode proteins rich in glycine, and LOC107816805 is commonly involved in biological processes such as cell wall formation and structural maintenance. It may act synergistically with GRP1 to confer cold stress resistance in tobacco. SRC2 in pepper has been found to synergistically induce defense responses against PcINF1 with SGT1, encoding a protein that forms protein complexes with receptor proteins [41]. These hub genes are activated by cold stress and exhibit higher expression levels in treatment A compared to lower expression levels in treatment B. This differential expression may be a primary factor contributing to the observed differences in cold tolerance between the two cultivation methods.

The Tan module exhibited high expression mainly after cold stress and three days post-recovery in treatment B. Hub genes within this module, such as RNP1, AGO1B, PP2AB2, and At4g33760, showed significant upregulation in both B1 and B2, with most of their functions related to RNA bioprocesses [42]. AGO1B is an important component in RNA silencing pathways, which can protect plants from RNA damage [43]. The large up-regulation of genes associated with RNA repair and adjustment following cold stress may suggest that the genetic material of B has been injured.

In our analysis, functions and genes related to energy conversion were predominantly identified in treatment A, significantly surpassing findings in other areas. Notably, this includes the AS gene, which regulates the synthesis of NAD precursors, the PED1 gene, which is closely associated with fatty acid β-oxidation, and the RROP1 gene, which is involved in ATP production. These findings suggest that the energy conversion capability of the shallow water seeding cultivation treatment is superior to that of the float system. This discrepancy in energy conversion efficiency may underlie the observed differences in cold tolerance between the two treatments under dark conditions following exposure to cold stress at 4 degrees Celsius.

## Conclusions

In summary, shallow water seeding cultivation demonstrates enhanced cold tolerance in tobacco seedlings compared to the float system. It has a more developed root system and higher antioxidant enzyme activity after encountering cold stress. On a deeper level, the shallow water seeding cultivation treatment may enhance the energy conversion capacity of tobacco seedlings by increasing the efficiency of NAD synthesis. The next step in our research will be to functionally validate these genes, whether the tobacco seedlings will maintain the appropriate cold tolerance after knocking out a certain gene. However, this discovery offers valuable guidance for exploring improved tobacco cultivation techniques.

## Materials and methods

### Plant material

Xiang Yan 7 is a flue-cured tobacco variety developed by the Hunan Tobacco Research Institute in China. It has been recognized as an excellent variety due to its strong disease resistance [44]. In comparison to other flue-cured tobacco varieties such as K326 and Yunyan 87, Xiang Yan 7 demonstrates superior yield and quality. For our experiment, we have opted to use seeds enclosed in artificial coating to ensure uniform conditions.

### Experiment design

**Fig. 12:**
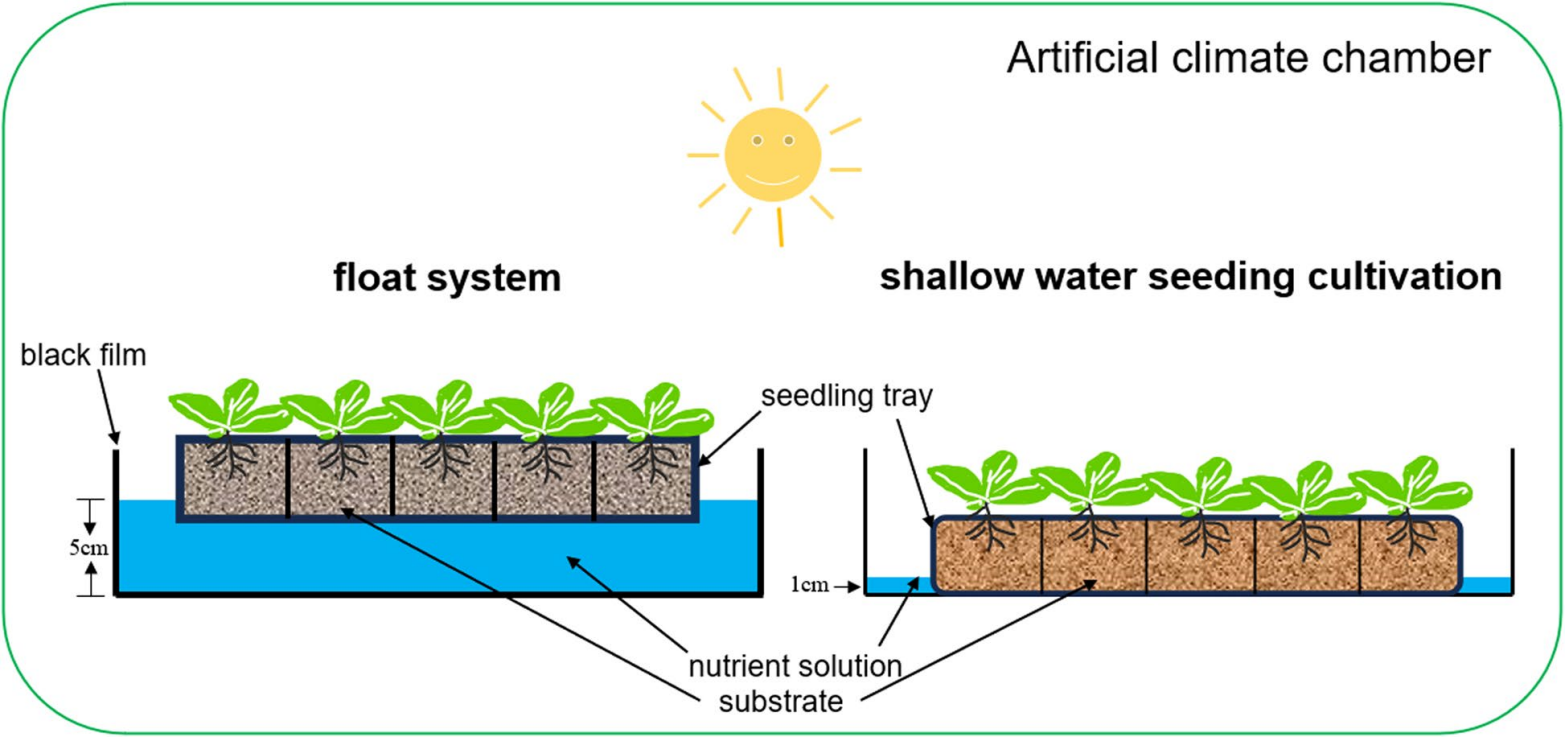
Schematic diagram of different seedling cultivation methods

To investigate the effects of different seeding cultivation methods (shallow water seeding cultivation and float system) on tobacco cold resistance, we conducted two treatments (Fig. 12): shallow water seeding cultivation (Treatment A) and float system (Treatment B).

The two treatments were conducted using the same sterile stainless-steel basin (dimensions: length 90 cm, width 60 cm, height 6 cm). The nutrient solution is prepared by dissolving powdered nutrients in water, with a ratio of 1 g of nutrient powder dissolved in 1 L of distilled water. The content and proportion of various components in the nutrient solution are as follows: (N: P_2_O_5_: K_2_O: Ca: Mg = 16:16:32: 2:1). The experiments were carried out in a greenhouse with 40% relative humidity, a 16-h light/8-h dark cycle, and a constant temperature of 25 °C.

Treatment A: Seedling trays are filled with an appropriately elastic seedling substrate and placed into a sterile stainless-steel basin. Each seeding tray is then sown with tobacco-coated seeds, and a small amount of water is sprayed onto the surface to facilitate seed coating cracking. Subsequently, a prepared nutrient solution is poured into the stainless-steel basin, allowing the nutrient solution to submerge the bottom of the seedling tray by 1 cm. This ensures that the substrate naturally absorbs the nutrient solution. For the initial absorption, the substrate is allowed to fully absorb the nutrient solution. During the experiment, 2L of nutrient solution is added to the stainless steel basin when a day after the nutrient solution was absorbed and evaporated.

Treatment B: A sterilized stainless-steel basin was used as a nursery pool, with its bottom and sides covered with black film. Nutrient solution was injected into the basin, filling it up to half of its height. Seedling trays had already been sown, with each hole containing a tobacco pill-coated seed. This ensured that the substrate fully absorbed the nutrient solution until saturation was achieved. The nutrient solution was then continuously injected until the liquid surface reached a height slightly lower than that of the seedling tray, at which point the injection was stopped. This method followed the tobacco seeding protocol with the float system (*GB/T 25241.1–2010, *https://openstd.samr.gov.cn/bzgk/gb/index).

At the 4-leaves-1-heart stage, healthy and uniform tobacco seedlings from both treatments were selected and exposed to a cold and dark environment (4℃, 80% humidity) for 12 h to induce cold stress. Subsequently, the seedlings were returned to the greenhouse for a 3-day recovery period. Plant leaves were collected using liquid nitrogen and stored in a -80℃ refrigerator.

All results presented in this study are based on the average of three independent biological replicates.

### Observation of root structure and detection of phenotypic data

The root growth of tobacco seedlings was photographed and documented at the stages of 2, 3, and 4 true leaves. For each treatment, representative and healthy tobacco seedlings were selected. The substrate was carefully rinsed off the root system with water. The roots were then spread out on a non-reflective black cloth using tweezers and photographed in a well-lit environment.

The method for detecting phenotypic data of root systems is as follows: At the stage of 4 true leaves, select well-grown and representative tobacco seedlings from different treatments. Remove the aerial parts, leaving only the root system. Carefully clean the substrate off the roots, then place the roots in a petri dish containing 5 mm deep distilled water. Use tweezers to fully spread out the roots in the water, ensuring minimal overlap of the primary roots. Place the petri dish into the WinRHIZO root analysis system for automatic detection.

### Physiological and biochemical indexes analysis

During cold stress, plants produce high levels of reactive oxygen species (ROS), which can disrupt normal cellular functions and lead to cell death. To investigate the impact of two different nursery methods on tobacco seedling cold resistance, we analyzed the activities of the antioxidant enzymes superoxide dismutase (SOD), peroxidase (POD), and catalase (CAT), as well as the malondialdehyde (MDA) content.

SOD was detected using the “Nitro Blue Tetrazolium (NBT) Method”. Prepare 0.05 mol/L phosphate buffer (PBS, pH 7.8), 14.5 mM methionine, 30 μM EDTA-Na2, 60 μM riboflavin, and 2.25 mM nitroblue tetrazolium (NBT) solutions. Homogenize 0.2 g of fresh plant sample in 1.6 ml of PBS, centrifuge, and use the supernatant as the enzyme extract. Mix the reaction solution and add the enzyme extract. Incubate the mixture under light for 20 min, then measure the absorbance at 560 nm. Calculate SOD activity based on the inhibition of NBT photoreduction, with one unit defined as the amount of enzyme causing 50% inhibition.

POD was detected using the “Guaiacol Method”. Mix 0.1 M phosphate buffer (pH 6.0) with guaiacol and H2O2 to prepare the reaction solution. Add 20 μl of enzyme extract to 3 ml of reaction solution in a cuvette. Measure the absorbance change at 470 nm every minute for 2 min. Calculate POD activity based on the rate of absorbance change, expressed as ΔA470 per minute per gram of fresh weight (ΔA470/min·g·FW).

CAT was detected using the “Hydrogen Peroxide Method”. Prepare a reaction solution by mixing 0.1 M H2O2 with 0.1 M phosphate buffer (pH 7.0). Add 0.1 ml enzyme extract to 2.5 ml of this reaction solution. Measure the absorbance decrease at 240 nm every minute for 2 min. Calculate CAT activity based on the rate of absorbance change (ΔA240/min·g·FW).

MDA was detected using the “Thiobarbituric Acid Method”. Prepare the reaction solution by dissolving 0.6 g TBA in a small amount of 1 M NaOH, then dilute with 10% TCA to 100 ml. Mix 1 ml of enzyme extract with 2 ml of 0.6% TBA solution, heat in a boiling water bath for 15 min, then cool quickly and centrifuge at 4000 rpm for 10 min. Measure the absorbance of the supernatant at 600 nm, 532 nm, and 450 nm. Calculate MDA content using the formula:

$$\text{MDA}(\text{nmol}/\text{g}\cdot \text{FW})=(6.45\times (\text{D}532-\text{D}600)-0.56*\text{D}450)\times 0.015/\text{W}$$

These parameters were assessed at three stages: before cold stress, after 12 h of cold stress, and after 3 days of recovery.

### Transcriptome sequencing

The transcriptome encompasses all RNAs transcribed by a particular tissue or cell during a specific time or under certain conditions, comprising primarily mRNAs and non-coding RNAs [45]. Transcriptome sequencing, conducted using the Illumina sequencing platform, investigates all mRNAs transcribed by a specific tissue or cell within a defined timeframe [46]. This approach forms the foundation for exploring gene function and structure and holds significant relevance in comprehending the development of organisms and the onset of diseases [47].

#### 1. Sample extraction and library QC testing

RNA was extracted from tobacco leaf tissue stored in a -80 °C refrigerator, and its integrity was verified using an Agilent 2100 bioanalyzer. Subsequently, library construction commenced with total RNA, wherein mRNA with a polyA tail was enriched using Oligo(dT) magnetic beads. The enriched mRNA was then randomly fragmented using divalent cations in the Fragmentation Buffer. Following this, the first strand of cDNA was synthesized using the fragmented mRNA as a template and random oligonucleotides as primers in the M-MuLV reverse transcriptase system. The RNA strand was degraded by RNaseH, and the second strand of cDNA was synthesized using dNTPs in the DNA polymerase I system. The double-stranded cDNA was purified, end-repaired, A-tailed, and ligated to a sequencing adapter. Subsequently, cDNA fragments ranging from 370–420 bp were size-selected using AMPure XP beads, amplified by PCR, and the PCR products were purified again with AMPure XP beads to obtain the final library. The constructed libraries were quantified using a Qubit 2.0 Fluorometer, and their insert size was confirmed using an Agilent 2100 bioanalyzer. Once the insert size was validated, the effective concentration of the libraries was accurately quantified using qRT-PCR, ensuring a higher concentration than that obtained with AMPure XP beads.

#### 2. Transcriptome sequencing and data quality control

Sample libraries that met the criteria were subjected to sequencing using Illumina technology. The sequenced fragments were then transformed into sequence data, or reads, through CASAVA base recognition of the image data generated by the high-throughput sequencer. These reads undergo filtering to retain only those with adapters, those containing undetermined base information, and those with high quality (reads with Qphred scores ≤ 5 bases, constituting 50% of the total read length). Following this filtering process, clean reads are obtained for downstream analysis.

#### 3. Mapping to the reference genomes

The genome sequence of Nicotiana tabacum (Cultivar TN90) was acquired from NCBI. These gene sequences were sourced from the NCBI database (https://www.ncbi.nlm.nih.gov/datasets/genome/GCF_000715135.1/). As genome sequences may differ between cultivars, this can lead to inaccurate or missing gene localization on the reference genome, and this may affect subsequent studies on biological functional pathways [48]. Before selecting TN90 as the reference genome, we analyzed the mapping rates of other Nicotiana tabacum cultivar genomes in the NCBI database. Ultimately, only TN90 met the required mapping rate criteria. Subsequently, an index of the reference genome was generated using HISAT2 v2.0.5. Paired-end clean reads were aligned to this reference genome using HISAT2 v2.0.5 for comparison and further analysis.

#### 4. Quantification of gene expression

The read counts mapped to each gene were computed using levelfeatureCounts (version 1.5.0-p3). Following this, levelfeatureCounts calculated the FPKM (Fragments per kilobase of exon model per million mapped fragments) for each gene, taking into account the gene's length. FPKM is a widely used method for estimating gene expression levels as it considers both sequencing depth and gene length, thereby providing a normalized measure of gene expression [49, 50].

#### 5. Differential expression analysis

Differential expression analysis between two comparison groups was performed using the DESeq2 software (version 1.20.0). DESeq2 employs statistical procedures based on the negative binomial distribution to identify genes showing differential expression in numeric gene expression data. The resulting p-values were adjusted using the Benjamini and Hochberg method to control the false discovery rate [51]. Genes with adjusted *p*-values ≤ 0.05, as determined by DESeq2, were classified as differentially expressed.

#### 6. GO and KEGG enrichment analysis of differentially expressed genes

The clusterProfiler R package was used to perform Gene Ontology (GO) and Kyoto Encyclopedia of Genes and Genomes (KEGG) enrichment analysis on the differentially expressed genes [52].

#### 7. Weighted correlation network analysis

WGCNA (Weighted correlation network analysis) is a systematic method used to explore gene associations across samples, aiming to identify synergistically changed gene sets and potential biomarkers or therapeutic targets. The R package WGCNA provides functions for weighted association analysis, enabling network construction, gene screening, cluster identification, topological feature calculation, data simulation, and visualization [53]. WGCNA is suitable for multisample data, typically requiring more than 15 samples. Input files include sample information, represented as a matrix with numeric traits, and gene expression data, commonly using FPKM values for transcriptome sequencing.

#### 8. qRT-PCR assay

The extracted total RNA was utilized for qRT-PCR assay, and after the RNA purity was detected to meet the requirements, reverse transcription was performed to form the cDNA strand, which was processed assay after completion. Selection of Ntubc2 as an internal reference gene [54], The results were output as relative expression levels after 2^−ΔΔCT^ operation [55]. The primer sequences involved we have uploaded in the additional file (Table S4).

### Statistical method

We used IBM SPSS 25 statistical analysis software for ANOVA and stats analysis.

### Supplementary Information

Supplementary Material 1.

Supplementary Material 2.

Supplementary Material 3.

Supplementary Material 4.

## Data Availability

RNA-seq data that support the findings of this study have been deposited in the Sequence Read Archive of National Center for Biotechnology Information (https://www.ncbi.nlm.nih.gov/) with the bioproject accession numbers PRJNA1113540. Search the accession code you can find the RNA-seq I uploaded. The project title is Nicotiana tabacum cultivar:Xiangyan 7 Transcriptome or Gene expression.

## Abbreviations

ROS: Reactive Oxygen Species
SOD: Superoxide Dismutase
POD: Peroxidase
CAT: Catalase
MDA: Malondialdehyde
NAD: Nicotinamide Adenine Dinucleotide
NADPH: Nicotinamide Adenine Dinucleotide Phosphate
ATP: Adenosine Triphosphate
WGCNA: Weighted Gene Co-expression Network Analysis
A: Shallow Water Seeding Cultivation Treatment
B: Float System Treatment
FPKM: Fragments Per Kilobase of Exon Model Per Million Mapped Fragments
KEGG: Kyoto Encyclopedia of Genes and Genomes
GO: Gene Ontology
DEGs: Differentially Expressed Genes
BP: Biological Process
CC: Cellular Component
MF: Molecular Function
